# John Haven Emerson (1906–1997): The Ultimate Pioneer of the “Iron Lung”

**DOI:** 10.7759/cureus.69964

**Published:** 2024-09-22

**Authors:** Tao An Chen, Wen Chung Hung

**Affiliations:** 1 Division of Respiratory Therapy, Department of Chest Medicine, Show Chwan Memorial Hospital, Changhua, TWN; 2 Department of Critical Care Medicine, Show Chwan Memorial Hospital, Changhua, TWN

**Keywords:** historical vignette, iron lung, mechanical ventilator, polio, respiratory care

## Abstract

John Haven “Jack” Emerson (1906-1997) was an American inventor who made significant contributions to the field of respiratory therapy. He is best known for his improvements to the iron lung, resulting in Emerson’s iron lung, which became a vital treatment for polio patients during the epidemics of the 1950s, saving countless lives. Over his career, Emerson held 35 patents and developed numerous innovative devices. He also contributed to the design of flow valves for the U.S. Army, diving equipment, and hyperbaric oxygen therapy tanks. Emerson’s forward-thinking approach led him to be among the first to recognize high-frequency ventilation as an effective technique. Despite not completing high school, his ingenuity earned him recognition from the American Thoracic Society, where he became the first non-physician to receive such an honor.

## Introduction and background

Early life and education

John Haven “Jack” Emerson was born on February 5, 1906, in New York City to a scholarly family [1-3]. He received his education in private schools, attending the Ethical Culture School as a young boy. Although he completed his studies there, he was missing a few required subjects, which led him to frequently joke, “I never graduated from high school,” as recalled by his son George. Jack’s father, Haven Emerson, was a Public Health Professor at Columbia University in New York [1]. This also had an important impact on John Haven Emerson's future.

## Review

Early career

At the age of 22, despite his family’s objections, Emerson acquired the basic equipment of a machine shop from the estate of a local inventor. He relocated this machinery to a small warehouse where he established his workshop. There, Emerson crafted custom research apparatuses for professors and researchers at renowned medical and physiology schools in the Boston area. In 1928, Emerson designed a Barcroft-Warburg apparatus for studying tissue respiration. Initially used for photosynthesis research, these devices later became essential tools in cancer studies [1].

Opportunities during the polio period

In the early twentieth century, polio epidemics became increasingly severe in the United States. The 1916 outbreak was the most devastating the world had seen at that time, with 27,000 cases reported nationwide, including 8,900 in New York City alone [4]. During the poliomyelitis epidemics of the last century, hospitals were overwhelmed with patients suffering from acute respiratory failure [5]. Emerson’s father, who was the Health Commissioner of New York City at the time, began to notice the rising number of polio cases. He pulled his son aside and suggested, “If you are ever going to make an artificial respirator, now is the time” [1,2]. But at that time Jack had to face a strong competitor, Philip Drinker [1,6].

The iron lung

The earliest body-enclosing respirator was described by Alfred F. Jones of Lexington Kentucky in 1864 [7]. The barospirator, first described in 1924 by Swedish physician-scientist Torsten Thunberg, was used as a ventilator to assist polio victims with breathing. Thunberg’s barospirator, a fully encasing device, was an early predecessor of the iron lung [8]. In 1929, Harvard engineer Philip Drinker (1894-1972) and pediatrician Charles F. McKhann III (1889-1988) reported using a mechanical respiratory device to support a young polio patient, which later became known as the iron lung [6,8]. Drinker subsequently sold his patent rights [6], and in May 1929, Drinker and McKhann published an article about iron lung and poliomyelitis [9]. Further studies included case reports demonstrating the successful use of the machine, notably a trial involving 30 polio patients, which was published the following year in The New England Journal of Medicine [10,11]. The medical establishment recognized the therapeutic potential of the Drinker respirator.

Emerson’s Improved Iron Lung

**Figure 1:**
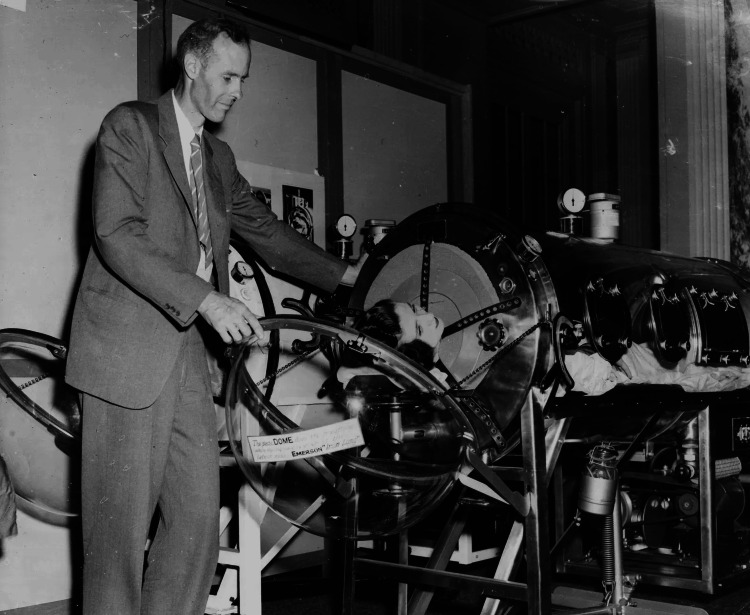
John Haven Emerson and Emerson’s iron lung. Image Credit: Permission obtained from Keystone Hulton Archive Getty Images [12].

Despite the groundbreaking benefits of the iron lung, it still had its flaws. Taking the opportunity to communicate with his father, and in another conversation with a friend who was a resident at Children’s Hospital, he asked if I had any ideas on how to improve the Drinker respirator. John Haven Emerson recalled that it was a new invention, with a price of around $3,600. It was a rectangular tank with thick, flat walls. The device had valves, motors to drive the valves, and blowers to change the pressure, which resulted in a lot of noise [2]. Emerson’s improving design introduced a flexible diaphragm to replace the blowers and valves used in the original model. This diaphragm was made from dual layers of elk hide, ensuring that if one layer tore, the second would continue to function. Additionally, Emerson refined the chamber’s shape and size, commissioning a boiler company in Boston (Market Forge) to manufacture it. The result was a device that was quieter, simpler, lighter, and more affordable (Figure 1) [1,2]. This improvement made Emerson’s iron lung more than half the price of the Drinker respirator [2,3]. This move brought tremendous benefits to society and polio patients at the time.

Battle of the iron lung machines

At the time, Drinker was the leading manufacturer of iron lungs [6]. Upon hearing about Emerson’s foray into respirator development, Drinker warned him that he held the patents for such devices and cautioned that litigation would follow if Emerson continued [6]. This situation exemplifies the spirit of Jack Emerson, showcasing his resilience and determination [1-3,6]. Eventually, he improved and produced an iron lung that was quieter, more reliable, and cheaper than Drinker’s. When the lawsuit was filed, Emerson and his colleagues embarked on an extensive review of engineering and medical literature from both Europe and the United States. Their research culminated in a bright yellow pamphlet, available from the J.H. Emerson Company [7], which includes photographs and drawings of negative pressure ventilation devices that predated Drinker’s for several decades [5,7,8]. Drinker’s patents were declared invalid due to a lack of originality in the invention [1,6]. Thus, it serves as a rare example of how an individual outside the field of medicine, like Emerson, made a significant contribution to medical progress during the polio era or pandemic.

Subsequent career

Over his career, Emerson held 35 patents and developed numerous innovative devices, including the first volume ventilator, an adult ventilator with intermittent mandatory ventilation (IMV) mode, oxygen tents, humidifiers, resuscitators, and mechanical ventilators. He also contributed to the design of flow valves for the U.S. Army, diving equipment, and hyperbaric oxygen therapy tanks. Emerson’s forward-thinking approach led him to be among the first to recognize high-frequency ventilation as an effective technique [1-3].

**Figure 2:**
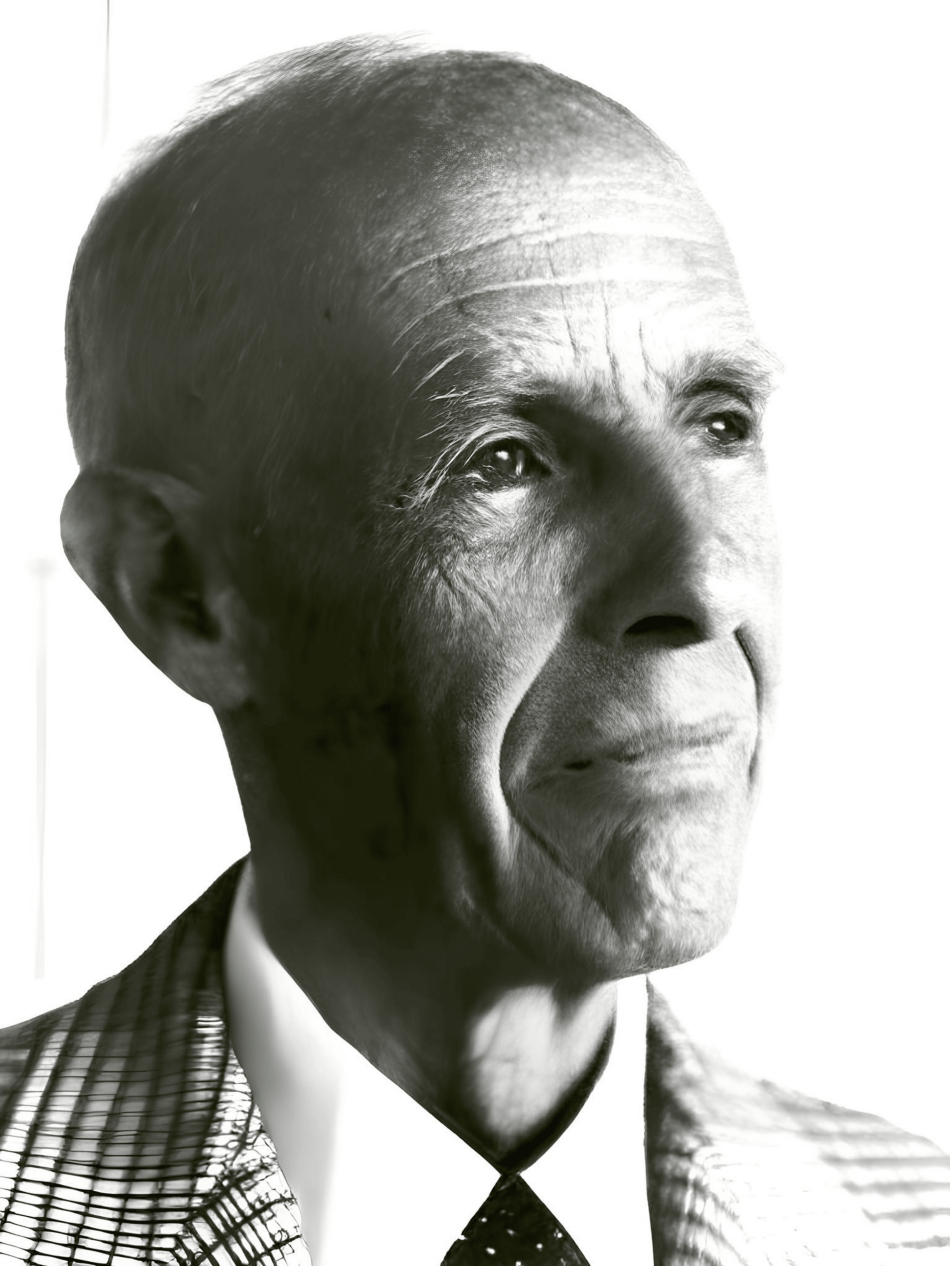
: John Haven “Jack” Emerson in his later years. Image Credit: Permission obtained from Litfl [13].

Emerson (Figure 2), widely known as “Jack,” was a trailblazer in the development of biomedical devices, with a strong focus on respiratory equipment [1,3]. When he passed away in February 1997 at the age of 91, it marked the end of an extraordinary and storied career, characterized by his quiet yet brilliant innovations [1].

## Conclusions

John Haven Emerson is recognized as a pivotal figure in the development of the “iron lung,” a mechanical respirator that played a crucial role in treating patients with respiratory failure, particularly during the polio epidemics of the early 20th century. His innovations and improvements to earlier designs made the iron lung more effective, affordable, and widely available, solidifying his legacy as the final and most significant contributor to its development.
